# The NSSI Family Distress Cascade Theory

**DOI:** 10.1186/s13034-018-0259-7

**Published:** 2018-12-18

**Authors:** Lisa Waals, Imke Baetens, Peter Rober, Stephen Lewis, Hanna Van Parys, Eveline R. Goethals, Janis Whitlock

**Affiliations:** 1 Vrije Universiteit Brussel, Brussels, Belgium; 2 Katholieke Universiteit Leuven, Leuven, Belgium; 3 University of Guelph, Guelph, Canada; 4 Universiteit van Gent, Ghent, Belgium; 5 HARVARD Medical school, Boston, USA; 6 Cornell University, Cornell, USA

**Keywords:** Nonsuicidal self-injury, Self-harm, Parental secondary stress, Family life cycle, Cascade model

## Abstract

Nonsuicidal self-injury (NSSI) is a complex behaviour and occurs most commonly during adolescence. This developmental period is characterized by the drive to establish an equilibrium between personal autonomy and connectedness with primary caregivers. When an adolescent self-injures, caregivers often experience confusion about how to react. Reports of feeling guilt, fear, and shame are common in the wake of learning about a child’s self-injury. This cascade of negative feelings and self-appraisals may lead to hypervigilance and increased caregiver efforts to control the child’s behaviour. The adolescent may experience this as an intrusion, leading to worse family functioning and increased risk of NSSI. This cascade is not well acknowledged or articulated in current literature. This article remedies this gap by presenting the NSSI Family Distress Cascade.

## Introduction

Non-suicidal self-injury (NSSI) is defined as the direct, deliberate destruction of one’s own body tissue without suicidal intent [1] and includes behaviors such as cutting, burning, and hitting oneself. The risk for engaging in NSSI is particularly high in adolescence, in which the onset is consistently found to be around 14-years of age [2, 3] and lifetime prevalence rates are about 17% in community samples [4]. Engagement in NSSI is strongly associated with various adverse mental health outcomes such as low self-esteem, depression, anxiety, and suicide attempts [5, 6]. Accordingly, it is unsurprising that the most common function of NSSI reported in non-clinical samples of adolescents is regulation of difficult and intense thoughts and feelings. Several recent studies [7, 8] suggest that NSSI in adolescence is a predictor for depression, anxiety, and suicide attempts later in life. Taken together, NSSI represents an important public health concern for today’s youth.

In line with its functions, in the emotional cascade model [9], NSSI is described as a result of a ruminative process (to an emotional stimulus) which results in a cascade which gradually increases emotional intensity and, ultimately leads to emotional dysregulation. The emotional cascade model asserts that NSSI serves as a form of distraction which temporarily reduces negative emotion and increases a perception of relief or even wellbeing. In this way NSSI represents a negative reinforcer in the emotion–behavior interaction. A more recent NSSI functional model, namely, the Cognitive-Emotional Model of NSSI [10], suggests that a number of cognitive processes also play a role in this emotional cascade to reinforce the behaviour. Importantly, the new model underpins the complex associations between cognition, emotion and behavior at an individual level. In another conceptual model, Hooley and Franklin [11] take into account some interpersonal factors (such as abuse/maltreatment/victimization and peer NSSI) in addition to the above factors. However, these interpersonal factors are only considered the level of triggers for NSSI. Notwithstanding the importance of these models, a conceptual framework to understand the interaction between NSSI and the environment is absent in current literature. The current paper presents a theoretical framework to understand the interaction between NSSI and the caregiver/adolescent relationship, which can be a first step to understand the interaction between NSSI within a broader context.

### The interaction between NSSI and the caregiver–adolescent relationship

Beyond its well-documented effects on those who self-injure, NSSI also has a significant impact on entire (family) systems [12–14]. After discovering a family member self-injures, most families experience acute stress and a sense of crisis. When caregivers find out about their child’s NSSI, they often feel overwhelmed and experience myriad emotions (e.g., anger, fear, guilt, confusion) [12–15]. Indeed, the impact of NSSI on caregivers through secondary stress/distress, can disrupt family dynamics and impede family functioning [16, 17].

In the (cross-sectional) studies examining the relation between NSSI and family processes to date, there exists a clear negative association between NSSI and a variety of family factors. For example, studies examining family functioning from the adolescent perspective, find that youth who self-injure report less emotional support, more criticism, and excessive behavioral control from family members [18] when compared to youth who do not self-injure. Furthermore, adolescents who self-injure also report being less securely attached to their caregivers [19]. Indeed, it is not uncommon for adolescents who self-injure to view their relationship with caregivers as unreliable and to believe that they may not be worthy of care. They also report difficulties integrating experiences across multiple levels of thinking and feeling (e.g., they may have a harder time adopting different perspectives), and difficulty forming reciprocal and empathic relationships with their caretakers [20].

The negative association between a variety of family factors and NSSI found in cross-sectional (adolescent-reported) studies is often interpreted causally. However, in the context of longitudinal and multi-informant studies, a more nuanced interaction between NSSI and the family emerges. For example, findings from longitudinal studies [12] show a dynamic and reciprocal pattern of influence between a child’s NSSI and parenting. Specifically, longitudinal research suggests that NSSI elicits more controlling (e.g., rule setting) parenting behaviors, which, in turn, is associated with more severe engagement in NSSI [12]. To this end, NSSI seems to impact the whole family system and can push a family system into crises.

In multi-informant studies, extant literature suggests that having a child who self-injures has a clear, and often adverse, impact on caregivers [14, 15, 21]. Indeed, researchers have suggested that managing care for a child who self-injures can result in “secondary stress” that is characterized by difficult thoughts and feelings (e.g., guilt, worry, or judgement) about the source of stress (such as a child who self-injures) or even oneself. This, in turn, can detrimentally impact daily life (e.g., logistical, emotional, attitudinal) [22].

Having a child who engages in self-injury can raise particular challenges as it tends to be episodic and thus difficult to anticipate [14]. As a result, caregivers of youth who self-injure often end up feeling overwhelmed or unable to manage their child’s needs [14, 15]. This, in turn, can lead to “empathy burnout” in which a parent becomes increasingly unable to respond in a compassionate way [23]. Chronic secondary stress can exacerbate self-injury duration and, in turn, reinforce negative family or parenting dynamics [18, 24]. For example, in a qualitative study by McDonalds, O’Brien, and Jackson [15], found that after NSSI was disclosed, mothers of children who self-injure reported high levels of perceived loneliness as well as fear of judgement by others in their peer groups (e.g. other mothers and fathers). Such feelings can inhibit reaching out to others, thereby increasing social isolation [12, 14, 16].

Confusion about why a child self-injures (and general misunderstanding about NSSI) can also affect parents. For instance, caregivers report feelings of insecurity, guilt, shame and a sense of personal responsibility for the fact that their child self-injures [12]. Furthermore, a lack of support coupled with a poor understanding of NSSI can hinder both the connection and effective communication with the child [14, 18, 24]. Moreover, fear about future episodes of NSSI or a suicide attempt can be paralyzing for caregivers who feel like they are “walking on eggshells” in anticipation of a future negative event that they cannot predict or control [15]. Finally, chronic emotional, social and practical (e.g., financial) strains can further compound the manner by which caregivers interact with and support their child [25].

The aforementioned cascade of aversive feelings and self-appraisals, along with confusion about how to best respond to their child, may lead to hypervigilance and increased efforts to control their child [12]. On the one hand, this control may be a way to deal with their own fear and insecurity. On the other hand, this may be perceived as intrusive by adolescents and may further strain family dynamics and increase the risk of NSSI [3]. To date, this family dynamic cascade is not acknowledged or well described in current NSSI literature. To address this, we present “The NSSI Family Distress Cascade.”

## The NSSI Family Distress Cascade Theory

This NSSI Family Distress Cascade Theory is interwoven with the psychosocial stages of development of adolescents (Identity vs. Role Confusion; [26]) and family life cycle stages of families with children in adolescence.

**Fig. 1:**
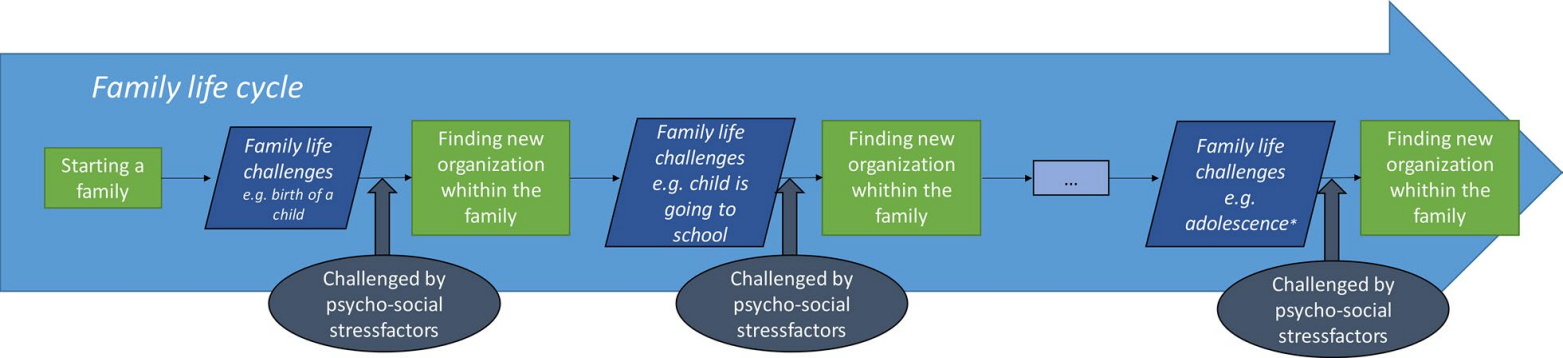
Family life cycle

Adolescence stands out among other stages in the family life cycle for its association with what can feel like tectonic shifts in family dynamics. Family equilibrium can be elusive in this period, largely because it is a period marked by dynamic tension between adolescent needs for both autonomy and connectedness. This can be challenging in and of itself, but is exacerbated by emotional and hormonal changes within the child and, often, a lack of understanding among caretakers of normative adolescent emotional and social development. As can be seen in Fig. 1, these factors may impede finding a new equilibrium within the family.

When normal shifts and challenges in family functioning are combined with mental health challenges, such as NSSI, family wellbeing can flounder. Since adolescence is the most common age of onset for NSSI [27], it is common for family systems to be impacted. Since adolescents sometimes injure themselves as a means to obtain relief from feelings of insecurity, self-doubt, and distress [1], the function of NSSI can interact with normative developmental drives and make parenting even more challenging. Caregivers, in particular, are often hard pressed to understand and navigate complex interactions while also setting effective policies and norms that work for everyone.

**Fig. 2:**
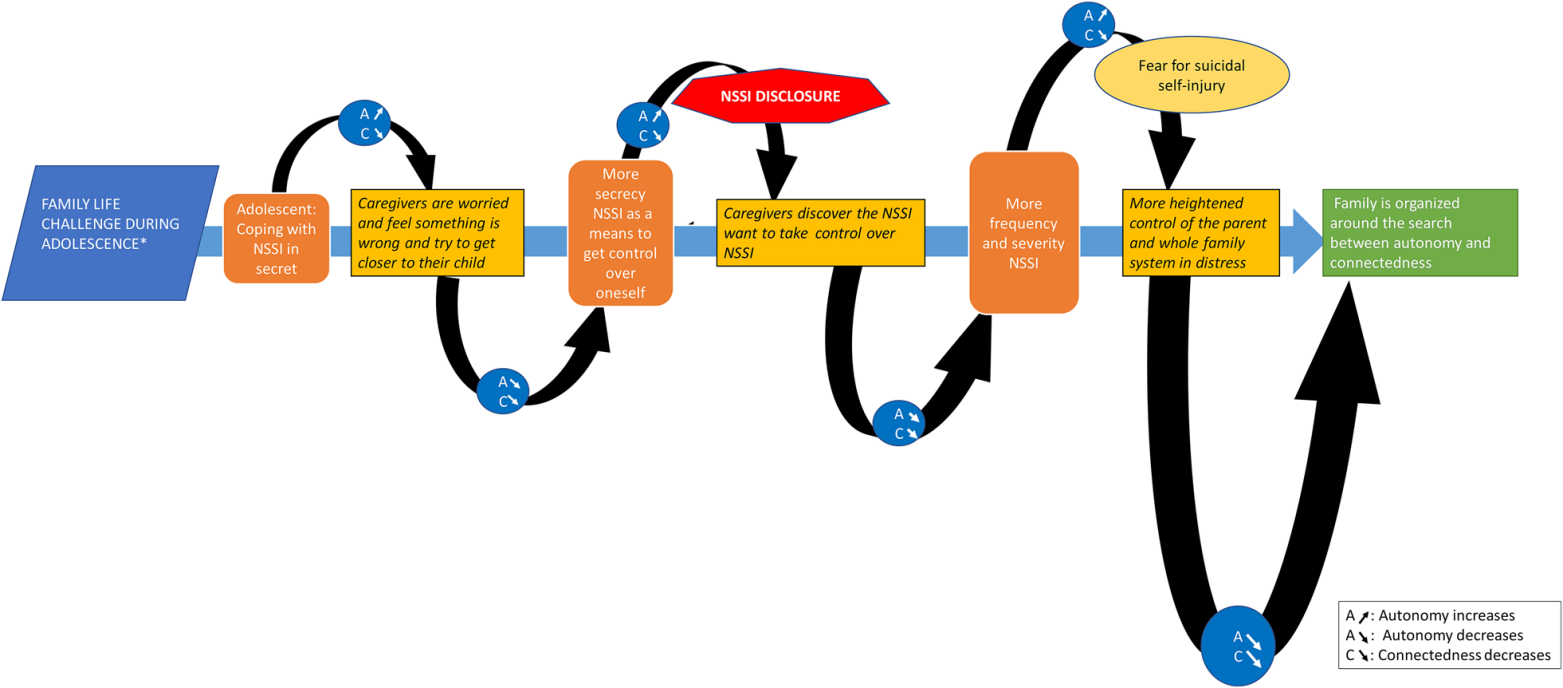
NSSI during adolescence finding an equilibrium between connectedness and autonomy

In the same way that there are patterns in family life cycles over time, the introduction of NSSI into a family system can be patterned enough to be described as a framework. As can be seen in Fig. 2, in the first stage, it is common for adolescents to keep NSSI hidden from their primary caregivers (e.g. [28, 29]), which may contribute to a sense of autonomy for the youth. Indeed, many adolescents describe NSSI as a symbol of autonomy [19]. Such secretiveness, however, is often noticed by caregivers who are well acquainted with the youth. While caregivers may not know what the youth is experiencing specifically, they often suspect that something is amiss (e.g., due to changes in social engagement or mood). At this stage caregivers may start to ask questions with the aim of understanding the source of their worry or as a means of reassuring themselves that there is not real cause for worry. These questions, though often well-intended, can be experienced as threatening to adolescent autonomy, which may compromise the sense of connectedness a youth has with the parent. This can exacerbate the need for secrecy and control which, in turn, increases parental suspicion, worry, and their pursuit to understand what is happening with their youth. In other words, the more secrecy on the part of the adolescent, the more caregivers may amplify their efforts to determine the source of stress.

Once this cycle starts, it can be difficult to interrupt. When NSSI is disclosed, parents often react with controlling behaviors (e.g., checking text messages or grounding their child) [24, 28, 29]. Adolescent may see these reactions as intrusions of privacy and overbearing parental hypervigilance [29, 30] as well as a threat to their autonomy. In turn, this may result in increases NSSI frequency and severity. Furthermore, in these times of family distress, the levels of stress, negative emotions and feelings of guilt in adolescents increase. To this end, the resultant cascade can further adversely impact NSSI.

The abovementioned escalating processes between the youth and their worried caregivers can be referred to as *complementary schismogenesis* [31] or *complementary escalation* [32]. Both concepts have been described in the family systems literature to describe the development of problems or pathology (e.g. [32]). A common feature of these processes is that each of the participants involved view their behaviour as the only sensible reaction to the behaviour of the other. In addition, both participants tend not to be aware of the overarching pattern in which they push the other to do exactly what they do not want the other to do. In the context of NSSI, the adolescent is in search of autonomy but pushes the caregivers to control him/her even more (e.g., *How can I decide for myself when mom and dad tell me what to do? How can I be myself if they know everything about me?*). In the case of caregivers, they want reassurance that their child is doing fine, but they push their child into more secret self-injuring behaviour as their only means to have a sense of autonomy. The caregiver’s persistent search for reassurance that their child is doing fine, may be understood by the adolescent as evidence that the caregivers do not want their child to be autonomous or that this is not valued. Indeed, youth may perceive their parents’ efforts as a means of control. Ultimately, this feeds adolescents’ resolve to not give in, and to guard their privacy (including how he/she deals with their body). This is consistent with recent research indicating that there is a positive association between NSSI and perceived parental control and identity issues [18], and a negative association between NSSI and perceived care [33].

In summary, on an individual level, NSSI offers relief. It is often a way of coping with the feelings of insecurity, self-doubt, and distress. On a family level, NSSI often plays a part in the adolescent’s search for identity and autonomy, within an attachment relationship.

## Discussion: ideas for future research and treatment

In this paper we propose a different way of looking at the crisis within families in which a young person self-injures. Specifically, we propose a cascade theory that highlights the dynamic and reciprocal relationship between the adolescent who self-injures and the family.

Despite many hypotheses about familial risk- and protective factors in NSSI, few studies have examined the bi-directional relationship between NSSI and family functioning. Most research uses retrospective methods, as well as adolescent-reported measures on potential familial risk factors. Although self-report approaches provide insight into adolescent perspectives, longitudinal multi-method designs (including parent-report data) are necessary for disentangling the interactive aspects of youth and caregiver factors since they elucidate the views of all parties involved. For example by using cross-lagged models, the interaction between caregivers reactions on NSSI and adolescents NSSI behaviors can be examined. Or to clearly map the *complementary schismogenesis* (*i.e. the escalating processes*) *as described in* NSSI Family Distress Cascade Theory momentary assessment can shed light on the *complementary escalation of both parent and child.* By researching multiple informants over a period of time one can begin to unravel the complexities and examine the role of negative cognitive biases of adolescents, and parental secondary distress after discovery of NSSI. To align the treatment for NSSI with these interactional processes between the needs of families and the needs adolescents who self-injure, qualitative research is needed to explore the experiences of adolescents who self-injure and their family with therapy, what are the helping factors and what are the hindrances they occur when seeing a therapist,…

## Towards a therapeutic answer

The NSSI Family Distress Cascade Theory, underscores the important role of systems and family therapies in NSSI treatment. It clearly suggests that family therapy, focusing on the strengths and resources of the child and their family within a systems context, may have utility in helping family members to better understand the nature of the patterns in which they may be caught. Furthermore, a family therapist can help family members to fully experience their love and care towards each other.

We expect different outcomes in systemic therapy than solely the cessation of NSSI. Systemic therapy focusses on the strengths and resources of the child and their family within the system. This may help family members to have a better understanding of the nature of patterns in which they might be caught. Furthermore, a family therapist can help family members to express their love and care towards each other. Research is needed to specify and identify actionable factors in the systemic approach of the adolescent who engages in NSSI and their family, in order to develop family interventions that are more tailored to the specific needs of each adolescent and their families. For example, the more cost-effective multi-family group interventions [34] may provide ample opportunities for caregivers and adolescents to discuss NSSI and its meaning for adolescents as well as for their families. In multi-family group intervention, adolescents as well as caregivers additionally benefit from the support of other adolescents and caregivers. Integrating a psycho-educational component in these interventions for caregivers on age-appropriate and qualitative involvement and communication with their adolescent about the NSSI might be meaningful.

## Conclusion

Within this paper we highlighted the importance of looking at NSSI and family dynamics in the context of a reciprocal process that impedes equilibrium in the family. While we apply this theory to NSSI in particular here, we expect that the NSSI Family Distress Cascade Theory has salience for a variety mental health challenges that families face. We also surmise this model having relevance for emerging adults. Understanding this model in the context of different caregivers may also have merit. For example, Hilt and colleagues suggest that mothers and fathers react differentially. In particular, they indicate that the father-child relationship will be more influence by situational factors, compared to the mother–child relationship. Further research, however, is needed to better understand these patterns and how this may fit within the currently proposed model.

Taken together, the current the NSSI Family Distress Cascade model may not only have empirical and clinical germanenes in the context of NSSI, as discussed above, but may also have applicability and relevance across a range of contexts.
